# Exercise motivation, barriers and HIIT preferences among Chinese college students: a qualitative study

**DOI:** 10.3389/fpubh.2026.1754702

**Published:** 2026-05-18

**Authors:** Cui Wendi, Li Hao, Tang Qi-Kai, Nor M. F. Farah, Arimi Fitri Mat Ludin

**Affiliations:** 1 Center for Healthy Ageing & Wellness HCARE, Faculty of Health Sciences, Universiti Kebangsaan Malaysia, Kuala Lumpur, Malaysia; 2 Nan Hang Secondary School, Nanjing, China; 3 Center for Community Health Studies ReaCH, Faculty of Health Sciences, Universiti Kebangsaan Malaysia, Kuala Lumpur, Malaysia

**Keywords:** barriers, Chinese college students, HIIT, motivation, physical fitness, preferences

## Abstract

**Introduction:**

The declining physical fitness of Chinese college students has become a major public health concern. To address this issue, this study serves as a preliminary needs assessment for the development of a tailored high-intensity interval training (HIIT) module. Guided by Self-Determination Theory (SDT), this study aims to explore exercise motivations, perceived barriers, and HIIT preferences among Chinese college students, providing a theoretically grounded understanding to inform intervention design.

**Methods:**

A qualitative study was conducted using non-probability purposive sampling to recruit Chinese undergraduate students aged 18 to 24. Semi-structured in-depth interviews were carried out, focusing on exercise motivations, barriers, and HIIT preferences. Data collection continued until thematic saturation was achieved. All interviews were audio-recorded, transcribed, and analyzed using a combination of inductive and deductive thematic analysis with NVivo 12. The inductive approach was used to explore participants’ exercise motivations and perceived barriers, allowing themes to emerge directly from the data. In contrast, a deductive approach was applied to categorize HIIT preferences according to six predefined dimensions based on established exercise guidelines. While themes were primarily derived inductively, their interpretation was informed by SDT constructs, including intrinsic and extrinsic motivation, amotivation, and the psychological needs of autonomy, competence, and relatedness.

**Results:**

Sixteen participants were included in the analysis. Five themes related to exercise motivation were identified: health benefits, self-interest, improved physical appearance, suitable training environment, and having training partners. These reflected different types of motivation, including intrinsic motivation (e.g., enjoyment), extrinsic motivation (e.g., health and appearance), and relatedness needs. Barriers to exercise were categorized into internal and external factors. Internal barriers included lack of motivation, bodily discomfort, lack of time, high perceived intensity of HIIT, lack of knowledge, and fatigue, many of which reflect amotivation or competence-related challenges. External barriers included academic pressure, environmental constraints, and physical condition. Regarding HIIT preferences, most participants preferred bodyweight-based exercises performed three times per week, with sessions lasting over 30 min.

**Conclusion:**

This study highlights that exercise behaviors among Chinese college students are shaped by varying types of motivation and the satisfaction or frustration of basic psychological needs as outlined in SDT. Supporting autonomy, competence, and relatedness may enhance engagement in HIIT and improve adherence. These findings provide a theoretical and empirical foundation for developing a tailored HIIT module that aligns with students’ motivational profiles and preferences.

## 1 Introduction

Regular participation in physical exercise is widely acknowledged as a fundamental strategy for promoting health and preventing disease (1). However, insufficient engagement in physical activity has become an increasingly prominent concern among young people globally (2). In particular, the physical fitness levels of Chinese college students have shown a continuous decline, accompanied by an increase in sedentary behaviors and limited involvement in structured exercise programs (3). Recent evidence indicates that 75% of university students in China fail to meet recommended physical activity guidelines (4). Furthermore, only 4.8% of students achieve the recommended level of moderate-to-vigorous physical activity (5). Although China places strong emphasis on promoting the physical health of college students, the outcomes remain suboptimal. Approximately 30% of college students nationwide fail to meet the standards of physical fitness tests, and problems associated with poor health—such as declining vision and increasing obesity rates—continue to persist (6). Many students demonstrate weak performance in strength and endurance assessments, including pull-up and long-distance running tests, with some unable to complete even a single pull-up or finish the required distance. Furthermore, a considerable proportion of college students exhibit postural problems, such as rounded shoulders, hunchback, scoliosis, and anterior pelvic tilt (7).

These health issues are closely linked to behavioral determinants, particularly insufficient physical activity and low exercise engagement. Previous research suggests that exercise participation among college students is strongly influenced by factors such as motivation, perceived barriers, and personal preferences (8). Despite awareness of the benefits of exercise, many students struggle to maintain regular physical activity due to competing academic demands, lack of time, and low intrinsic motivation. Therefore, understanding these behavioral determinants is essential for developing targeted and effective exercise interventions.

Exercise plays a crucial role in promoting physical health and overall well-being. It is an effective means to address obesity and increase lean body mass (9). Regular physical activity is inversely associated with the risk of numerous chronic diseases, including cardiovascular disease, diabetes, hypertension, and certain types of cancer (10). Among college students, exercise has also been shown to alleviate chronic pain—those who engage in physical activity more frequently, for longer durations, or at higher intensities report a lower risk of chronic pain and fewer pain sites (11). Moreover, exercise contributes to improved sleep quality and helps mitigate symptoms of depression and anxiety (12). It also enhances various aspects of physical fitness, including muscular strength, endurance, cardiovascular function, and flexibility (13). Beyond physical benefits, exercise supports cognitive health, improving both overall cognition and subcognitive domains, and can help counteract cognitive decline (14). Given that insufficient physical activity is one of the major contributors to global morbidity and mortality (15), it is imperative to promote physical activity and develop effective strategies that encourage college students to engage in regular, efficient, and sustainable exercise to improve their health outcomes.

High-intensity interval training (HIIT) has emerged in recent years as a popular and effective exercise modality that integrates both aerobic and anaerobic components. The core principle of HIIT involves alternating short bouts of high-intensity exercise with brief recovery intervals. This structured pattern of exertion and rest rapidly elevates heart rate, activates major muscle groups, enhances cardiovascular function, promotes fat oxidation, and improves muscular strength and endurance. HIIT has gained widespread acceptance and popularity due to its efficiency, time-saving nature, and ability to enhance oxygen uptake and metabolic rate. Moreover, the high-intensity nature of HIIT induces an “excess post-exercise oxygen consumption (EPOC),” whereby the body continues to expend energy and burn calories even after the exercise session concludes, facilitating sustained fat metabolism and weight management (16).

Given the multiple benefits of high-intensity interval training (HIIT), integrating it into the daily physical exercise routines of college students is both scientifically sound and innovative. However, despite the well-established effectiveness of HIIT in improving physical fitness, its successful implementation among college students is not guaranteed. Research has shown that exercise participation is strongly influenced by individuals’ motivation, perceived barriers, and personal preferences (17). Many students, although aware of the benefits of exercise, struggle to maintain consistent participation due to lack of motivation, academic pressure, and time constraints. Therefore, before developing a customized HIIT training module for Chinese college students, it is essential to conduct a systematic needs assessment to ensure that the program design aligns with their actual needs and behavioral tendencies.

This study is guided by Self-Determination Theory (SDT) (15), a widely applied framework for understanding motivation in physical activity contexts. SDT distinguishes between intrinsic motivation (engaging in an activity for inherent enjoyment), extrinsic motivation (engaging for external outcomes such as health or appearance), and amotivation (lack of intention to act). Central to SDT are three basic psychological needs, autonomy (a sense of volition), competence (a sense of effectiveness), and relatedness (a sense of social connection) which influence the quality of motivation and behavioral persistence.

SDT has been extensively used to examine exercise behavior, including among university students and in high-intensity interval training (HIIT) contexts, where motivation plays a critical role in adherence and engagement (18, 19). Therefore, SDT provides an appropriate theoretical lens to explore college students’ exercise motivations, barriers, and preferences, and to inform the development of tailored HIIT interventions.

In this context, the present study aims to explore Chinese college students’ exercise motivations, perceived barriers, and preferences related to HIIT, thereby providing essential insights for the future development of an effective, engaging, and sustainable HIIT module tailored to this population.

## 2 Materials and methods

This qualitative study aims to explore the motivations, barriers, and preferences regarding HIIT training among Chinese college students from Heilongjiang Province. This study employed a semi-structured in-depth interview with the aim of comprehensively obtaining participants’ actual perspectives. The study adhered to the Consolidated Criteria for Reporting Qualitative Research (COREQ) checklist to ensure comprehensive and transparent reporting. The interview guide used in this study was developed based on a previous study (20). A pre-test was conducted with three students before the formal interviews, and the interview script was revised based on the pre-test results. All interviews were conducted in Chinese by Cui Wendi (CWD). To minimize interviewer bias, a standardized interview guide was used, and the interviewer followed neutral, non-leading questioning techniques. Open-ended questions and consistent procedures were applied across all interviews to ensure reliability.

The interview script was divided into two parts: the first part concerned the participants’ exercise habits, and the second part focused on their knowledge of and attitudes toward HIIT training. Participants who exercised regularly were asked about their motivations for regular exercise and the barriers they faced. Those who did not exercise regularly were asked about the reasons for not exercising and how they might develop an exercise habit. Next, participants were asked whether they had heard of HIIT training. Those who had heard of HIIT were asked about their knowledge of HIIT, their motivation to engage in HIIT training, and the barriers to practicing it. For those who had not heard of HIIT, the interviewers provided a brief introduction before asking about their motivation to engage in HIIT and the barriers they might face. All participants were asked about their preferences regarding HIIT, such as the type, frequency, duration, and location of the training.

Each interview lasted approximately 10 min and was conducted between May to July 2025 at the Qiqihar University campus. Although the interview duration was shorter than that of traditional in-depth qualitative interviews, the interviews were highly focused and guided by a structured interview protocol targeting specific domains (motivation, barriers, and HIIT preferences). The relative homogeneity of the study population (Chinese college students with similar academic and lifestyle contexts) enabled participants to provide concise yet relevant and information-rich responses. Importantly, data saturation was achieved, as no new themes or codes emerged in the final rounds of interviews. This suggests that the depth and comprehensiveness of the data were sufficient to address the research objectives.

### 2.1 Sampling and recruitment

We followed the operationalization of (21), setting the initial interview sample size to 10. After completing these 10 interviews, we analyzed the interview results. If new material continued to emerge, we conducted three additional interviews. This process was repeated in sets of three until no new material appeared, which would be considered the point of data saturation. After the 13th interview, no substantial new themes were identified, and this pattern remained consistent in subsequent interviews, indicating that thematic saturation had been reached. This approached is supported (22) where they found that saturation was achieved between 5 to 24 interviews. We employed a non-probability purposive sampling method and recruited 16 undergraduate students from Qiqihar University in Heilongjiang Province. The inclusion criteria were a) Formally registered as a student at Qiqihar University during the research period; b) Aged between 18 and 24 years old. Students who were unable to safely participate in physical activity or had conditions that could significantly influence their exercise behavior beyond the scope of this study were excluded. Recruitment and interviews continued until data saturation was reached.

### 2.2 Data analysis and processing

This study employed both inductive and deductive thematic analysis, with each approach applied to different components of the data in a complementary manner (23). Inductive thematic analysis was used to explore participants’ exercise motivations and perceived barriers. In this approach, themes were generated directly from the data without imposing pre-existing theoretical structures, allowing participants’ lived experiences to guide theme development. Although an inductive thematic analysis approach was adopted, the interpretation of themes was informed by Self-Determination Theory (SDT), allowing for a theoretically grounded understanding of participants’ motivations, barriers, and preferences in relation to autonomy, competence, and relatedness. In contrast, deductive thematic analysis was applied to the analysis of HIIT preferences. Specifically, participants’ responses were categorized according to six predefined dimensions (type, frequency, duration, timing, location, and social context), based on the ACSM Guidelines for Exercise Testing and Prescription. This theory-driven approach enabled a structured examination of exercise preferences aligned with established exercise science frameworks. The integration of both approaches allowed for a comprehensive understanding of the research problem, combining data-driven insights with theory-informed categorization.

All interviews were recorded in video format using smartphones, then transcribed and manually proofread with the Feishu Miaojia software. The qualitative analysis software NVivo 12 was used to analyze the data thematically, identifying participants’ motivations for and barriers to HIIT training (24). The data were analyzed using data triangulation (25). In the first step, the transcribed data were uploaded to NVivo 12 and coded by two members of the research team (CWD and LH). The codes were then consolidated according to the research questions. To ensure coding reliability, the two researchers independently coded the data and subsequently compared their coding results. Any discrepancies were discussed and resolved through consensus. When necessary, a third researcher was consulted to reach agreement. The second step involved reviewing the coding by the other two researchers. After comparison, the codes were consolidated into broader categories, and the relevant themes were identified. The third step was integrating the themes. Finally, all researchers held discussions to finalize the codes, categories, and themes. Regarding preferences for HIIT training, participants’ answers were categorized into six predetermined themes: type, frequency, duration, timing, location, and whether the training was done individually.

To enhance the methodological rigor of this qualitative study, several strategies were employed. First, inter-coder reliability was strengthened through independent coding by two researchers (CWD and LH), followed by iterative discussions to resolve discrepancies and reach consensus. Although Cohen’s kappa coefficient was not calculated, this consensus-based approach is widely used in qualitative research to ensure coding consistency. Second, an audit trail was maintained throughout the analytical process, documenting coding decisions, theme development, and data interpretation to ensure transparency and traceability. Third, deviant or negative cases were actively examined during analysis to capture diverse perspectives and avoid overgeneralization of findings.

### 2.3 Validity and reliability

To ensure the rigor of this qualitative study, several strategies were employed. First, researcher triangulation was applied, whereby multiple researchers were involved in coding and reviewing the data to enhance credibility. Second, discrepancies in coding were discussed and resolved through consensus, ensuring consistency in the analytical process. Third, an audit trail was maintained throughout data collection and analysis to enhance transparency and dependability. Finally, reflexivity was considered during the research process, with the researchers remaining aware of and minimizing potential subjective influence on data interpretation.

### 2.4 Ethics statement

This study has obtained ethical approval from the National University of Malaysia relevant institutional review board before the commencement of the study (Ethics Ref. No.: JEP-2025-206). Respondents were briefed on the study purpose and procedures. Consent forms were emailed to individual respondents, who signed and returned the forms prior to the interviews, ensuring confidentiality and anonymity throughout the research process.

### 2.5 Strengths of the study

This study has several strengths. First, the use of a qualitative design allowed for an in-depth exploration of college students’ exercise motivations, barriers, and preferences, providing rich and context-specific insights. Second, the study focused specifically on Chinese college students, a population that has received relatively limited attention in qualitative HIIT-related research. Third, by examining motivations, barriers, and preferences simultaneously, the study offers a comprehensive understanding of factors influencing exercise behavior. These findings provide a valuable foundation for the development of tailored HIIT interventions for this population.

### 2.6 Limitations

This study has several limitations that should be acknowledged. First, the participant recruitment was from a single institution (Qiqihar University) may limit the transferability of the findings to broader populations. In addition, as with all qualitative research, the data are subject to inherent self-report bias.

Although several strategies were employed to enhance analytical rigor, certain qualitative validation procedures were not implemented. Specifically, inter-rater reliability was not quantified using statistical measures such as Cohen’s kappa. However, coding consistency was ensured through independent coding and iterative discussions among researchers to reach consensus.

Furthermore, although both male and female participants were included, the study did not perform a systematic gender-based analysis, which may have overlooked potential gender-specific differences. Future research should address these limitations by including more diverse samples and incorporating more rigorous validation procedures.

## 3 Results

### 3.1 Respondents’ characteristics

A total of 29 participants were initially recruited for this study. However, data saturation was achieved after the 16th interview, and no new themes emerged. Therefore, a total of 16 participants were included in the final analysis. These 16 participants were aged between 18 and 24, all were Chinese, and were non-sports major undergraduate students from Qiqihar University. Table 1 summarizes the characteristic of the participants.

**Table 1:** Characteristics of participants.

Subject code	Gender	Age	Grade	Major
R1	Female	20	2	Marxism
R2	Female	20	2	Marxism
R3	Male	18	1	Geographic Information Science
R4	Male	18	1	Geographic Information Science
R5	Male	21	3	International Economics and Trade
R6	Male	21	3	International Economics and Trade
R7	Male	20	2	Mechanical Engineering
R8	Female	19	2	Chinese Language and Literature
R9	Female	20	2	Chinese Language and Literature
R10	Male	19	2	Material Science and Engineering
R11	Male	20	2	Material Science and Engineering
R12	Female	21	3	Computer Science and Technology
R13	Female	20	2	Computer Science and Technology
R14	Male	21	3	Electronic Information Engineering
R15	Male	20	2	Electronic Information Engineering
R16	Male	20	2	Electronic Information Engineering

### 3.2 Motivation to exercise

The motivations that drove respondents to participate in exercise were diverse. The themes identified reflect different forms of motivation and psychological needs as conceptualized within Self-Determination Theory. In this study, five themes were identified regarding the motivations of college students to participate in exercise: health benefits, self-interest, improved physical appearance, suitable training environment and having training partners. Details are shown in Table 2.

**Table 2:** Motivation to participate in exercise.

Theme	Subtheme
Health benefits	Improve immunity
	Improve physical fitness
	Improve psychological wellness
	Improve sleep quality
	Weight loss
Self-interest	
Improve physical appearance	
Have a suitable training environment	
Have training partners	

**Theme 1: Health benefits.** Fourteen respondents reported that exercise would benefit their health. When probed further, more than half highlighted improvements in physical fitness (*n* = 12). Other perceived benefits included weight loss (*n* = 6), improved immunity (*n* = 6), enhanced psychological well-being (*n* = 3), and better sleep quality (*n* = 3).

Twelve respondents participated in exercise to improve their physical fitness, as R5 mentioned:

*"It can enhance muscle strength, improve cardiovascular function, boost endurance and flexibility, thereby improving overall fitness” (R5, age 21).*

Six respondents mentioned that exercise helped them lose weight, as R10 stated:

*“I used to be overweight, and after starting running, I noticed a significant improvement in my cardiovascular health and weight loss" (R10, age 19).*

Another six respondents mentioned that exercise could, or they hope it would, prevent colds and boost immunity, like R5 said:

*“If I felt that it was particularly beneficial for my health, like improving my immunity, I'd start doing it” (R5, age 21).*

Health includes physical and mental well-being, and mental health is also very important for college students. Three respondents mentioned that exercise could improve their mood, as exemplified by R3, who said:

*"I think it improves my mental health, as exercise makes me feel happy and relieves stress, anxiety, and depression” (R3, age 18).*

Additionally, three respondents mentioned that exercise could improve their sleep quality.

**Theme 2: Self-interest.** Six respondents participated in the exercise based on interest.

*“If I enjoy the feeling of the exercise after trying it for a while, I'd do it regularly” (R14, age 21).*

*“Developing an interest in it. If I can turn it into a hobby, I’ll practice it regularly” (R14, age 21).*

Theme **3: Improved physical appearance.** Three respondents participated in the training to improve their physical appearance. University years coincide with adolescence, a period when students generally have higher expectations regarding their appearance.

*“Make my body more proportionate” (R2, age 20).*

*“I'd want to be healthier and improve my figure” (R9, age 20).*

Theme **4: Suitable training environment.** One respondent stated that having a suitable training venue was important.

*“There should be a suitable training venue that is not affected by the weather” (R6, age 21).*

Theme **5: Have training partners.** Seven respondents mentioned that having a training partner was *essential for motivating them to participate in exercise.*

*“If someone accompanies me to train, I might feel more motivated and willing to try. On my own, it’s much harder” (R11, age 20).*

*“Still working out with others- this could help me develop a habit” (R4, age 18).*

### 3.3 Barriers to exercise

Based on respondents’ answers, the barriers to engaging in exercise were coded into internal and external factors. Internal factors included lack of motivation, bodily discomfort, lack of time, perceived high intensity of HIIT, lack of knowledge and feeling tired from exercising, while external factors included academic pressure and environment, and physical condition. Details are shown in Table 3.

**Table 3:** Barriers to participate in exercise.

Theme	Subtheme
Internal	Lack of motivation
	Bodily discomfort
	Lack of time
	Perceived high intensity of HIIT
	Lack of knowledge
	Feeling tired from exercising
External	Academic pressure
	Weather conditions
	Inadequate sports facilities

**Theme 1: Internal.** Exercise participation among college students is influenced by multiple factors. These include lack of motivation (*n* = 8), bodily discomfort (*n* = 6), lack of time (*n* = 6), perceived high intensity of HIIT (*n* = 5), lack of knowledge (*n* = 3) and feeling tired from exercising (*n* = 3). Together, these factors shape students’ willingness and ability to engage in regular physical activity.

Respondents lacking motivation primarily exhibited behaviors such as avoiding exercise due to laziness, lack of interest, and poor mood.

*“It's probably occasional laziness, I just want to lie in bed and play on my phone, not exercise” (R16, age 20).*

*“Also, emotions play a role—sometimes feeling down makes me not want to move” (R11, age 20).*

Respondents who mentioned bodily discomfort face the following obstacles: physical and mental exhaustion caused by daily chores, fatigue or injuries resulting from exercise, and, for women, menstruation, which could also hinder participation in exercise training.

*“Occasionally, I also get injured while playing basketball, like spraining my ankle or straining a muscle, which can prevent me from exercising for a period of time” (R14, age 21).*

*“During my period, when I feel unwell, I tend to skip exercise” (R12, age 21).*

Respondents who cite lack of time as an obstacle all mentioned that daily tasks often took up the time meant for exercise. For the category perceived high intensity of HIIT, one of the respondents, R14 said:

*“The intensity of HIIT workouts is too high for me, and I will get very tired” (R14, age 21).*

Three respondents stated that they lacked the knowledge to create their own plans and correctly perform HIIT training. R6 said:

*“Another issue is that I might not know how to create a workout plan, and making one on my own could be quite challenging” (R6, age 21).*

Three respondents stated that the reason for not exercising was that it caused fatigue. As for fatigue after exercising, R2 said:

*“Because I feel that exercising makes me very tired, and my stamina can't keep up. I don't like this feeling of exhaustion and fatigue” (R2, age 20).*

Theme **2: External.** External factors were fewer compared to internal factors and primarily included three identified sub-themes: academic pressure (*n* = 6), weather conditions (*n* = 5), and inadequate sports facilities (*n* = 1). Academic pressure was a common challenge that college students had to face. As mentioned by R7:

*“Having a busy class schedule and homework after school takes up a lot of time and energy, which can hinder exercise” (R7, age 20).*

Five respondents mentioned that they might not be able to exercise due to weather conditions. One of the respondents said:

*“There are also weather-related reasons, such as not being able to play basketball outdoors on rainy days, and cold weather affecting exercise” (R5, age 21).*

One respondent stated that the lack of sports facilities hindered her from exercising (R1, age 20).

### 3.4 HIIT preferences

The concept of exercise preference refers to an individual’s tendency and liking for certain types of exercise or workout methods, based on factors such as interest, goals, health conditions, and more (26). Everyone’s exercise preferences may vary. We categorized respondents’ HIIT preferences into type, frequency, duration, time, location and alone or with others, based on ACSM’s Guidelines for Exercise Testing and Prescription (27). Type refers to the mode of exercise. Frequency indicates how often the exercise is typically performed within a week. Duration refers to the length of a single exercise session. Time refers to the time of day when the exercise is performed. Location specifies where the exercise takes place. Alone or with others refers to the preference for training alone or with multiple people. Details are shown in Table 4.

**Table 4:** HIIT preferences among respondents.

Theme	Subtheme
Type	Running, cycling, swimming (*n* = 5)
	Bodyweight training (*n* = 10)
	With equipment (*n* = 2)
Frequency	2 times/week (*n* = 3)
	3 times/week (*n* = 12)
	4 times/week (*n* = 1)
Duration	≥30 min (*n* = 11)
	<30 min (*n* = 5)
Location	Indoor (*n* = 9)
	Outdoor (*n* = 8)
Time	Morning (*n* = 5)
	Afternoon (*n* = 4)
	Evening (*n* = 11)
Alone or with others	Group training (*n* = 9)
	Individual training (*n* = 7)

For the types of HIIT, most respondents preferred bodyweight training (*n* = 10) while a few chose running, cycling, swimming (*n* = 5), or with equipment training (*n* = 2). In terms of frequency of HIIT training, most respondents preferred times a week (*n* = 12), while only a few preferred trainings twice (*n* = 3) or four times (*n* = 1) a week. For the training duration, more than half of the respondents preferred training sessions lasting 30 min or longer (*n* = 11), while a few preferred sessions under 30 min (*n* = 5). Regarding training locations, the number of respondents who preferred indoor (*n* = 9) and outdoor (*n* = 8) settings was roughly the same. As for training time, most people preferred exercising in the evening (*n* = 11), while a few preferred trainings in the morning (*n* = 5) or afternoon (*n* = 4). The number of respondents who preferred training alone (*n* = 7) was roughly equal to those who preferred training in groups (*n* = 9). These findings suggest that college students tend to prefer exercise formats that are flexible, accessible, and manageable within their daily schedules, while still being effective for improving physical fitness.

## 4 Discussion

This study explored Chinese college students’ exercise motivations, barriers, and preferences in the context of HIIT, interpreted through the lens of Self-Determination Theory (SDT). The findings highlight how different types of motivation (intrinsic, extrinsic, and amotivation) and the satisfaction or frustration of basic psychological needs (autonomy, competence, and relatedness) shape exercise participation.

Participants’ interest in exercise and enjoyment of physical activity reflect intrinsic motivation, which has been consistently associated with sustained engagement in physical activity. In contrast, motivations related to health benefits and physical appearance align with identified regulation, a more autonomous form of extrinsic motivation. These findings are consistent with SDT-based research suggesting that more self-determined forms of motivation are associated with greater adherence to exercise behaviors. Health-related benefits emerged as the primary motivation for exercise among participants, suggesting that college students place strong value on maintaining and improving their overall health. This finding may be explained by increasing health awareness and exposure to public health information, as well as growing concerns about declining physical fitness and lifestyle-related health risks in this population. In addition, the emphasis on outcomes such as physical fitness, immunity, and psychological well-being indicates that students tend to associate exercise with both physical and mental health benefits. This aligned with the findings of Iker and Lago’s research (28, 29). Previous studies have shown that the exercise motivation of college students can directly influence their physical fitness or affect it indirectly by influencing their physical activity (30).

According to a study on the exercise motivation of college students, it was found that students were generally motivated to engage in physical exercise for reasons such as appearance, strength, endurance, stress management, weight management, and overall health (31); this was similar to the findings of our study. College students generally possess relatively high energy levels and a strong desire to explore new things. As a result, a significant portion of them are eager to improve their physical fitness through exercise, enabling them to take on more challenges (32). A study from China indicates that poor academic performance is linked to poor physical fitness (33). Improving physical fitness can also help alleviate psychological anxiety among college students (34). Some college students who are passionate about sports aim to enhance their athletic performance by improving their physical fitness (7). Therefore, improving physical fitness is a particularly important motivation for college students to engage in sports.

Modern college students commonly face weakened immune function due to irregular routines (35), high stress levels (36), insufficient physical activity (2), and unbalanced diets (37). A compromised immune system increases the risk of infections and may reduce vaccine efficacy (38). Evidence suggests that regular exercise can enhance immune function and contribute to the prevention of various infections (39). This may explain why many participants identified health-related benefits as a key motivation for engaging in exercise.

In addition, lifestyle changes after entering university may further contribute to health concerns. Without parental supervision, many students adopt less structured routines, including excessive screen time, irregular eating patterns, and poor sleep habits (40, 41). These behaviors increase the risk of overweight and obesity, which are associated with adverse health outcomes such as abnormal metabolic indicators, fatty liver, and hypertension (42). Such risks may further reinforce the importance of exercise as a preventive strategy.

Mental health is another important dimension influencing overall well-being among college students. Depression and anxiety are among the most commonly reported psychological issues in this population (43), often linked to academic pressure, employment concerns, interpersonal relationships, and financial stress (44). Exercise has been shown to play a significant role in alleviating these symptoms (45), which helps explain why several participants reported mood improvement and stress relief as key motivations for engaging in physical activity.

Poor sleep quality is also prevalent among college students (46). In addition to irregular sleep schedules, psychological factors such as anxiety and depression can further contribute to sleep disturbances (47). As a result, some students may use exercise as a strategy to regulate sleep patterns and promote relaxation, thereby improving both physical and mental recovery.

The second motivation for respondents to take part in exercise was self-interest. Some respondents stated that regular exercise was one of their hobbies, while others mentioned that if they could develop an interest in a particular sport, they would be more likely to engage in it. A Norwegian study (48) indicates that college students’ interest in sports comes from three main aspects. First, they are motivated by fun and spending time with friends, which, on a broader level, helps them expand their social network. Second, competition and mastery play a significant role as competitive sports serve to measure both individual and team skill levels. Third, students appreciate how participating in sports positively impacts their everyday health. Cultivating an interest in sports requires a combination of personal experience, social interaction, and positive reinforcement. By creating a supportive environment, choosing suitable types of sports, and setting achievable goals, sports can gradually become an essential part of daily life, ultimately fostering long-term interest (49).

The third motivation is to improve physical appearance, which aligns with the findings of other studies reporting that college students engage in sports to enhance their appearance (50–52). Relevant research (53) found that the level of personal satisfaction with one’s body shape was strongly correlated with the degree of participation in exercise. Individuals with better body shapes tend to engage more in exercise whereas those who are dissatisfied with their body shape or have a less desirable physique are likely to suppress their exercise behavior. A positive body image among college students can enhance and foster stable self-esteem, which is a key factor in promoting more effective participation in sports (54). However, it is worth noting that many college students tend to focus on their appearance and body shape while neglecting their health (55). In today’s digital era, the widespread exposure to idealized body image can lead to low body satisfaction, which may, in turn, increase the risk of developing mental disorders (56). College students should actively participate in exercise and adopt scientific methods to improve their physiques and appearance, thereby promoting a healthy and positive form of beauty.

Two other key motivations are having a suitable training environment and training partners. Access to suitable sports environment significantly influences individuals’ motivation to engage in physical exercise. A study analyzing college students’ exercise motivations and influencing factors found that school sports environment was a primary dimension affecting students’ exercise motivation (57). Having exercise partners can significantly enhance an individual’s motivation to engage in physical activity. Research indicates that peer relationships, and exercise experiences positively influence the development of exercise habits (58). Additionally, studies have found that social support, including having regular exercise partners, positively affects college students’ adherence to exercise routines (59). The presence of workout partners can also improve college students’ self-efficacy, thereby promoting greater engagement in exercise (60).

Lack of motivation emerged as the most prominent internal barrier to exercise among participants, indicating that sustaining engagement in physical activity remains a key challenge for college students (61). This finding suggests that, despite awareness of the benefits of exercise, many students struggle to translate intention into consistent behavior. Several participants attributed their low motivation to perceived time constraints related to academic workload, as well as the absence of clear and achievable exercise goals. In addition, the lack of immediate results appeared to reduce persistence, highlighting the importance of outcome expectations in maintaining exercise behavior.

Another theme in internal barriers is lack of knowledge. Lack of exercise knowledge can lead to low exercise participation rates among college students, inability to engage in exercise correctly and efficiently, and lack of understanding about the importance of exercise for health. The promotion of exercise knowledge for college students should be taken seriously. Universities should organize more health and sports knowledge lectures, place more emphasis on physical education classes, and combine practice with theory. Diverse physical activities should be held on campus to cultivate students’ interest in sports. The role of physical education teachers should be highlighted, as they should not only teach sports skills but also serve as guides for students’ sports knowledge, fostering students’ health awareness during lessons.

Another internal barriers identified was inadequate sports facilities. There are indeed certain issues with public sports facilities in China. First, funding shortages: building public sports facilities often requires significant financial investment, but due to limited local government budgets, it is difficult to ensure adequate financial support (62). Second, poor planning: in some regions, insufficient consideration is given to factors such as population distribution and usage frequency during the planning process, resulting in uneven facility distribution or low utilization rates (63). Third, limited variety of facilities: in some areas, public sports facilities are mainly focused on one or a few types of sports, making it difficult to meet the diverse needs of the population (64). There are also common issues with sports facilities in universities, such as limited availability of venues and restricted usage time due to tight competition for resources. Many facilities are outdated and poorly maintained. Additionally, indoor facilities are insufficient, and during adverse weather conditions, the limited number of indoor venues cannot meet students’ needs.

We also collected respondents’ preferences regarding HIIT training, it provides novel insights into college students’ preferences for HIIT training, particularly in terms of exercise type, timing, and social context. The majority of participants reported a preference for engaging in HIIT sessions three times per week, whereas a smaller proportion indicated a preference for either two or four sessions weekly. Furthermore, most participants favored a training duration exceeding 30 min per session, while a minority preferred sessions lasting 30 min or less. These preferences meet the WHO global recommendation for physical activity, which recommend 75–150 min of vigorous-intensity physical activity per week (65), and the Chinese Ministry of Education and Sports Bureau’s recommendation of 1 h of physical activity per day and at least three times per week of moderate to vigorous intensity exercise (66).

First, bodyweight-based HIIT was the most preferred exercise modality among participants. This finding is consistent with previous research indicating that young adults tend to favor exercise modes that are convenient, accessible, and do not require specialized equipment (67). Bodyweight training reduces environmental and logistical constraints, making it easier to integrate into daily routines, especially for students with limited time and resources (68). Second, most participants expressed a preference for exercising in the evening. This aligns with existing studies suggesting that college students are more likely to engage in physical activity after completing academic tasks, as evening hours provide greater flexibility and fewer time conflicts (69). Circadian rhythm research also indicates that physical performance and perceived exertion may be more favorable later in the day, which could further explain this preference (70).

Third, preferences for exercising alone versus with others were relatively balanced. This reflects findings from previous studies showing that while some individuals value autonomy and flexibility in solo exercise, others benefit from social interaction, peer support, and accountability associated with group-based training. Social support has been consistently identified as a key factor influencing exercise adherence and motivation among college populations (71). Lastly, the respondents who prefer to do HIIT training indoors and outdoors are evenly split. In indoor training, the environment is controllable, unaffected by weather, and the temperature, humidity, and air quality are more stable. Indoor training venues like gyms are equipped with various specialized fitness facilities to meet diverse training needs. On the other hand, outdoor training is influenced by weather and seasonal changes, but on pleasant days with comfortable temperature, outdoor workouts are enjoyable. Compared to gyms, outdoor training relies more on the natural environment and outdoor equipment. Therefore, in the future, we will focus on developing HIIT training modules based on bodyweight exercises.

Among the barriers, some respondents mentioned that the high intensity of HIIT training prevented them from engaging in it. This is consistent with some previous studies (72–74). Despite the significant health benefits and the safety and feasibility of HIIT, the intensity of the exercise may become a barrier for some participants, who may feel concerned or fearful about its high intensity. Therefore, to address the barrier of perceived high intensity, several structured strategies can be implemented. First, HIIT programs should be individualized based on participants’ fitness levels and health conditions to enhance safety and accessibility. Second, a progressive training approach should be adopted, beginning with lower intensity and longer recovery intervals, followed by gradual increases in intensity and reductions in rest periods to support physiological adaptation. Third, incorporating engaging elements such as group-based training and gamified formats may help reduce perceived exertion and improve overall adherence.

Understanding the motivations and barriers to exercise among Chinese college students has given us a clearer insight into their exercise needs and the challenges they need to overcome. This is crucial for promoting physical activity among Chinese college students and improving their physical fitness. We have also gained an understanding of their preferences for HIIT training, which provides a foundation for designing and developing effective HIIT training modules for Chinese college students in the future.

## 5 Conclusion

This study identified five themes related to exercise motivation, including health benefits, self-interest, improved physical appearance, suitable training environment and having training partners. It also identified barriers to exercise, which were categorized into two main themes: internal and external factors. Internal factors include lack of motivation, bodily discomfort, lack of time, HIIT training intensity being high, lack of knowledge and feeling tired from exercising, while external factors include academic pressure and environment and physical condition. The study also found that most respondents preferred bodyweight exercises as their preferred type of HIIT training, with a frequency of three sessions per week, each lasting over 30 min, and some respondents reported being deterred by the high intensity of HIIT, these findings highlight the importance of developing tailored and feasible HIIT interventions that align with students’ preferences and address both psychological and practical barriers. Such approaches may improve participation and support the establishment of long-term healthy exercise behaviors among college students. Present study contributes to the literature by applying Self-Determination Theory to understand exercise motivation among Chinese college students in the context of HIIT. The findings emphasize the importance of supporting autonomy, competence, and relatedness in designing effective exercise interventions.

## Data Availability

The raw data supporting the conclusions of this article will be made available by the authors, without undue reservation.
